# The Province of Porcelain Work in Dentistry

**Published:** 1904-06-15

**Authors:** Hart J. Goslee

**Affiliations:** Chicago, Ills.


### THE PROVINCE OF PORCELAIN WORK IN DENTISTRY.

BY HART J. GOSLEE, D.D.S., CHICAGO, ILLS.

Read before the Ohio State Dental Society, Columbus, Ohio, December 2, 1903; and printed from advance proofs by courtesy of "Dental Summary."

The subject of porcelain work in the numerous phases of its application to dentistry is an important feature of the more modern practice of the profession of the day. The employment of porcelain encompasses so great a scope of general usefulness that it is undoubtedly destined to increase in attractiveness, and to continue to be so regarded in proportion to the more general development of desire for the accomplishment of higher artistic attainments.

Notwithstanding the truth of so reasonable an assertion and prophecy, it has only recently been said by a prominent writer that "the profession is being deluged with a tidal wave of enthusiasm for porcelain work, from the undertow or reaction of which it will be some time in recovering."

In my opinion this statement should be carefully digested, because it is both false and true—paradoxical though such an assertion may seem it is true only in so far as *over*-enthusiasm may induce an injudicious application; and it is equally false because there always will be a certain proportion of cases where the application may have been accidentally or otherwise confined within the range of its practical limitations.

A degree of enthusiasm in any special line of thought, or in any particular field of labor, whether it be of a scientific or practical nature, may react to the injury of the theory or its product, and yet it quite as often serves a useful purpose by the stimulation and encouragement which it engenders.

The wheels of progress in whatever line have doubtless gained more momentum from the activity and energy of the enthusiast than from the philosophy of the conservativest. If this is true—and a liberal review of the history of the progress of the world would seem to so indicate—then it must in turn be also true that we must never overlook, or fail to pay tribute to, even that class of enthusiasts whom some are disposed to designate as "cranks," because the conservatively-inclined are more or less dependent upon the cranks for the inspiration which stimulates and encourages the development of the more logical minds.

This applies to the general use of porcelain in dentistry in so far as that it warrants us in conceding that even the most radical enthusiast commands and deserves our respect and consideration for his vigorous efforts to encourage the adoption of methods of procedure which enables us to better serve our patients in the preservation or restoration of the teeth. That this class of men has done much to increase our artistic attainments, and incidentally has aided very materially in elevating the profession to a position where it commands the respect of the people, and where any insinuation that it is only the vocation of the artisan or craftsman can with justice and propriety be refuted, there can be no doubt.

Whilst I am willing to acknowledge a debt of gratitude to those whose overzealous enthusiasm has made them known as "cranks," but whom I prefer to call *radical enthusiasts*, it is from the efforts of the *conservative enthusiast* that has come the real *development* and *progress* of porcelain work, in its various phases, to its present degree of perfection.

In reviewing the progress and considering the present status of porcelain work, it will be found that the profession may be generally divided into two classes: Those who, at the expense of time and experimentation, and in the face of discouragements have become sufficiently familiar with its technical manipulation as to acquire a degree of confidence and skill which has enabled them to obtain, to a greater or less extent, the perfection of art which is possible; and, on the other hand, those who have been so influenced by the radical enthusiast to expect to achieve success without acquiring the skill or the fundamental knowledge that is a first essential. They have not been willing to apply themselves so as to be able to surmount obstacles and overcome discouragements and have thus met with such failures as might be expected to result from the efforts of those who have undertaken to work without a definite knowledge of fundamental principles. Without this experience and skill which others possessed they should not necessarily influence us or cause skepticism, or provoke condemnation. Indeed, no man does justice to his profession, or even to his own intelligence, who condemns that which he is not thoroughly familiar, or which may have failed in his hands until he has positively ascertained that the fault was not entirely his own.

It is these unreasonable and unwarranted expectations which account for a large proportion of failures, and which do more to retard progress than to advance it.

Since the advent of porcelain work, the atmosphere surrounding the practicability of its application has been impregnated with the skepticism, and even the cynicism, of a large proportion of the profession; and yet, notwithstanding such influences, it has developed into an *art,* and one which is surely destined to occupy a permanent and conspicuous place in the practice of dentistry of the future. It is an "art"' however, which requires so high a degree of skill and delicacy of manipulation in its application as to account for the occasional or frequent discouraging failures of some. We are not born skillful; this is something which must be acquired, and to develop it to the necessary extent to insure success means perseverance and application to a degree which too often men fail to recognize. The trouble with many of us is we forget that we must crawl before we can walk, and that we think that we *ought* to know before we really do know.

To appreciate the possibilities of this class of work and to be successful in its application, it is necessary that one should study the cause of failures. That one should possess a liberal knowledge of the underlying fundamental principles and requirements; should appreciate to the fullest extent the importance of detail, and be scrupulously painstaking in their execution; that he should possess the higher ideals of the artist, combined with the instincts of the mechanic, and above all, that he should possess that degree of discretion and good judgment which is so tersely expressed in that commonplace and homely phrase, "*horse-sense,*" is positively true.

While it is true that the advantages of porcelain work are largely of an esthetic nature, the hygienic properties afforded by its presence in the mouth are scarcely second in material importance and desirable significance. Although porcelain *is* a more or less porous substance, yet, notwithstanding a statement to the contrary recently made by a prominent writer, the *surface* of the well-vitrified piece of porcelain is practically impervious to the penetration and accumulation of secretions, which is not true of the surface of gold or platinum, or any of the metals, no matter how highly polished.

In considering the usefulness of porcelain or of any other substance or method of procedure it is the duty of the dentist to preserve the teeth, and it should be the effort of the modern practitioner to accomplish this: First, in a manner which offers the greatest possible degree of permanency; second, in a manner which will most closely simulate the natural, or, rather, the typically-normal condition as possible; and, third, in a manner which will offer the very highest degree of hygienic properties. I believe that the judicious and skillful use of porcelain offers the maximum of possibilities in all three of these cardinal prerequisites.

Formerly porcelain art work was confined to the construction of artificial dentures, and in this particular field of usefulness its application still offers the greatest possible opportunities for utilizing its many desirable qualities.

The same law governing general application also holds good in this, as in all other indications; hence the degree of discretion and judgment which has been mentioned must also be exercised in the selection of cases favorable for its application, if successful achievements are to result. It must be remembered that the employment of porcelain offers the highest degree of artistic possibilities, but that these do not constitute the entire scope of the requirements for successful operations. A conservative and judicious application, such as would insure success, would confine the indications for such dentures to that class of full upper cases where the mouth is in a more or less favorable permanent condition of absorption; and to that class of partial cases where a similar condition exists, and in both of which classes a sufficient bulk of porcelain to insure the required integrity may be employed. And then this must also be even further supplemented by a metal base which will afford adequate support and protection to the porcelain.

This is always necessary, because porcelain is a friable substance, and wherever its employment requires that it must sustain stress, it should be fortified against the dangers of such by a strong and rigid metal framework. Hence, its use would be contra-indicated in cases where such opportunities are not afforded, for this reason it is not so well adapted to the construction of lower dentures, because of their more or less unfavorable shape, and for the reason that other substances and other methods will better conform to one of the requirements by offering a greater possible degree of permanency.

This combined requirement of favorable absorption, and of metal construction, as applied particularly to the indications for and requirements of full upper dentures, has often been used as an argument against this class of work, the argument being based upon the possible displacement of the case in the mouth, owing to its weight.

As the retention of an upper denture is due mainly to an adaptation to tissues of an unequal resistance upon which it rests, which will afford an equalization of pressure upon both hard and soft places, and as this is then supplemented by the action of the muscles of both cheeks and lip, I agree quite fully with Doctor Haskell in that weight cuts but little, if any figure, providing such requirements have been skillfully observed.

A further application of porcelain to prosthesis, and particularly to the construction of partial dentures other than continuous gum, which will add materially to the artistic possibilities, may be observed in the making of gum blocks for individual cases. Such blocks of two or more teeth may be easily made by first selecting suitable facings; grinding them to the desired adaptation; soldering them together with a small platinum or platino-iridium wire; burnishing platinum foil to a close adaptation to the model, and filling in between it and the facings with porcelain and gum enamel until the desired gum restoration may have been produced, and then subsequently effecting the attachment to the base by whatever means may be indicated.

Also, in the construction of partial cases on any kind of base, where single teeth are to be supplied and where a more or less extensive absorption demands some effort toward an artificial reproduction of such tissue, a most artistic result may be obtained by selecting a tooth of the proper length to correspond with the adjacent teeth, and then extending its neck with sufficient gum enamel to reproduce the lost tissue and admit of proper contact with the gum. This may be done previous to grinding the tooth to its adaptation, and will then admit of a very artistic observation of the requirements in securing such contact, and of those incident to symmetrical alignment and corresponding length with adjacent teeth, all of which are so essential to artistic results, and yet so difficult to accomplish with the blocks or single gum teeth as procured from the supply house.

All that has been said with regard to the requirements in the application of porcelain for the construction of dentures is equally applicable to bridge-work. Personally, I am not much in favor of constructing *extensive* bridges in porcelain, because of its shrinkage in fusing, and friability afterward, combined with the difficulty of repair; and, yet, the artistic results possible, where the indications are favorable, where the requirements are carefully observed, and where the detail is skillfully executed, make for it a permanent place in the construction and application of either fixed or removable bridges.

The advent of platinum solder, or of an alloy for assembling the various parts of the platinum superstructure in a manner which precludes a possible disarrangement of individual relation in the subsequent fusing of the porcelain, has done much toward making successful results possible; and, if the application be confined to the construction of cases which are favorable, in accord with the previously mentioned opportunities and requirements, porcelain bridges up to the extent of five or six teeth at least, may be made with every assurance of success. All teeth included in the bridge, however, which are to possess masticating surfaces, and which are to withstand masticatory stress, must have such porcelain *adequately supported by the metal superstructure,* or failure will be sure to follow, owing to the brittle and friable nature of the porcelain.

In the construction of individual crowns, for the ten anterior teeth, or for those teeth which are within the range of vision, where the esthetic requirements are so great, there is positively no other method of procedure incident to their artistic and more or less permanent restoration which offers the same favorable opportunities.

This is particularly true of restoring the bicuspids where the glaring gold crown is so often seen, and quite as frequently regarded as the only means by which permanency may be insured; this is also true of the remaining anterior teeth. If a definite line of construction which will conform to the highest degree of mechanical as well as artistic requirements is observed, there is no doubt but that such crowns will possess a degree of inherent strength next to that of the gold crown, and exceeding that of my other style of construction.

The application of this art to the filling of teeth is the most modern phase of porcelain work, and marks an era in the progress of dentistry as a true art. The beautiful and artistic results which are possible command for porcelain in this particular application the respect of both the profession and the laity, and make for it a permanent and conspicuous place among the methods of arresting the progress of caries of the teeth, and restoring them to their original outline; and yet an injudicious and indiscriminate use has already caused its abandonment by some.

When employed in the manner indicated, nothing will serve the same purpose in all phases of the requirements, but where contra-indicated, other methods will doubtless serve to even much greater advantage.

In my opinion a judicious employment of porcelain inlay work would confine its application largely to approximal, labial or buccal and incisal or occlusal cavities, of favorable shape and proportions, in the eight or possibly ten anterior teeth, or, in other words, mainly to those cavities which are within the range of vision. But wherever it is necessarily subjected to undue masticatory stress, or where the cavity proportions, or opportunities for mechanical retention and resistance may be unfavorable, its use is contra-indicated, because other methods of procedure will usually offer greater opportunities for more permanent results.

In conclusion, I desire only to emphasize that, with a correct knowledge of the province and limitations of porcelain work, with the acquirement of skill in and the more conservative application of this art, and with the improved facilities in the nature of products and furnaces, the modern practitioner should be able, if his ambition so directs him, to conserve through this work the very highest possibilities of the dental art.

### DISCUSSION.

DR. H. C. RAYMOND, Detroit, Mich.: Dr. Byram has set before us very clearly his opinion of the relative values of high and low-fusing porcelain, and I congratulate him for the able manner in which he has carried out his subject. I cannot say whether it was also his object to give a paper in which there would be little or nothing to discuss, but if such was his intention, I congratulate him in the accomplishment of that also, for the paper contains very little for discussion. His treatment of the subject is somewhat in the nature of a general bird's-eye view, followed by a set of axioms framed to establish, or definitely settle, disputed points. But though an axiom is generally accepted as a self-evident proposition or fact, it is not necessarily so, and there are two or three statements laid down as facts by the essayist which I think are open to exceptions. Treating the subject as the writer has, in such a general way, his views will in the main be endorsed by those familiar with the work, and I heartily agree with much that he has said.

That porcelain inlay work is not a fad I am sure all will believe who have practiced it successfully, and there are many who are doing so, and there will be very many more doing so as time goes on and proves to us what a great tooth conserver it is. Though I am probably as enthusiastic about it as any one, I am not one who thinks that porcelain is going to entirely supplant gold and some of the other filling materials, at any rate not at present, for they all have their place, especially gold; but I do believe that the time is coming, if it is not already here, when the only excuse for using gold in anterior teeth, where it will mar the beauty of the face and sometimes disfigure it, will be because the patient can not afford porcelain, for unfortunately at present, the skill and time required to put in porcelain fillings precludes the possibility of using it for the masses. That, however, is nothing against porcelain itself. I think the time is not far distant when gold fillings in the anterior teeth will be looked upon with as much disfavor as are gold crowns in a like position. We can hardly tell from hearing the paper, whether its author inclines more to the high-fusing porcelain than to the low, or not. We might infer that he is on the fence, not at present having any choice in the matter I don't hesitate to say that I am a high-fusing porcelain worker, and I became one after trying and observing both methods. But I am not one who does not see any good in the low-fusing porcelain, for I know that those who are skilled in its use get some beautiful results. The question of relative strength of the two materials may, I think, be entirely dispensed with, for the high grades of either material is sufficiently strong for our purpose. From some tests I have made, I can not see that there is much difference between Jenkins' enamel and some of the high-fusing materials, though some very high-fusing porcelain, such as S. S. White's, is stronger than any low low-fusing porcelain. There is a great deal of talk about the density in one porcelain, and the translucency in another—one man saying that density is more requisite than translucency, and *vice versa*, according to the degree the particular porcelain he is interested in posesses these qualities. The natural teeth, we know, have a translucency; it is only slight, if any, at the necks of the teeth, but increases toward the incisal edge, where it becomes very marked owing to the bulk of the enamel there.

Now, we would naturally suppose that the nearer we can approach this translucency of nature where it exists the better we will succeed in imitating nature, and such, I believe, has been the experience of the most foremost porcelain workers Too much density gives in a degree opacity, and opacity gives a china-like appearance to the porcelain, and robs it of the life-like resemblance to the natural teeth. It is because the high-fusing porcelain is more translucent and life-like than the low-fusing that I place it above the latter in this respect. The necks of some teeth show considerable opacity or density, and in such cases the Jenkins porcelain with its greater density might serve the purpose better than the more translucent high-fusing porcelain. I myself, however, have found no difficulty with the use of the high-fusing material in such cases. The difference in the degree of polish that may be obtained after grinding the porcelain, the low-fusing men claim to be very much in their favor, but, as a matter of fact, there is so little difference in this respect that it would really not be worth referring to were it not that in this connection, I might say, owing to the greater tendency of low-fusing porcelains to bulge or assume a globular form in fusing, it nearly always necessitates grinding and polishing after the filling is set; whereas, the high-fusing porcelain retains its form almost perfectly, and if carefully built to the margins of the matrix requires little or no grinding or polishing when finished. The less we grind porcelain after the final fusing, the better, for no polish we can give it equals the glazed surface it has when taken from the furnace.

Dr. Byram says that, owing to statements made by some porcelain workers, many think that the cement is all that is necessary to retain a filling in position, and, judging from the number of fillings that come out, I think such must be the case. We must have a definite form for the cavity that will act as retention, as well as the cement to hold a filling. I don't mean anything in the nature of an interlocking form, as for gold, but a distinct cavity formation for porcelain, and this cavity formation is something that requires great care. We hear saucer-shaped cavities spoken of and written of by many, and some low-fusing advocates talk and write as if it was impossible to burnish a platinum matrix into any but a saucer-shaped cavity, pointing to that as a weakness or disadvantage in the use of platinum for the matrix. Such statements are very misleading, because they are untrue. I never form a saucer-shaped cavity. It would invite failure. All my cavities I give a decided form, and to some of them considerable depth. The inlay, however small, has always a decided seat to rest upon. It can only go into one position, and can not roll around when being set. I know of no cavity into which a gold matrix can be burnished that a platinum matrix can not also be burnished into. To repeat what I have written before, generally speaking, I can see but little if any advantage that the low-fusing porcelain possesses over the high-fusing, but do see advantages that the latter has over the former. The platinum matrix is easier to handle after removal from the cavity, and requires no investment, which a gold matrix does, excepting in the simplest cases. To say nothing of the time required for investment, to me the invested matrix is not so simple and easy to work with as the uninvested one, and besides I know of no investment material that will not change form under heat, and so may cause change in the form of the matrix and the finished inlay. We can not carve the low-fusing porcelain as we can the high. To me the method employed in high-fusing porcelain work is simpler and more definite than that used for low-fusing porcelain, though I don't think the question of time should enter in when we are working for artistic results. Since Dr. Byram referred to the difference in the time required between the two materials, in small fillings, I will say that the large contour fillings, owing to there being much less shrinkage in the high-fusing porcelain, and its ability to retain its form under heat, it takes up less time than does the low-fusing, as it requires fewer bakings to complete the filling. For the reasons mentioned, I am strongly inclined to favor the high-fusing porcelain, and I can strongly recommend its use to those starting out in the work, for I believe with it they will sooner obtain definite and pleasing results.

Although the electric furnace is the ideal one for porcelain work, the absence of the electrical current in the smaller towns need not deter any one from using the high-fusing porcelain, as there are small gasoline furnaces now on the market that will fuse almost any kind of porcelain. There will probably always be more or less difference of opinion regarding the relative merits of direct burnishing and working from impressions and casts of the cavity. For some compound cavities in bicuspids and molars, I start the matrix by swaging and make the final burnishing in the mouth. In the great majority of cases, however, I burnish directly into the tooth, and I consider it a simpler, quicker and more sure method of getting a perfect matrix.

I can not help referring to what Dr. Byram said regarding those who try to do this work in the mouth before mastering the technic involved. That is simply inviting failure and discouragement. One must become familiar with the entire process of making inlays before working in the mouth. We were not able to go and make a beautiful gold filling or perform some other difficult operation in the mouth just from seeing some one else do the work, but had to practice and labor hard to achieve such results. Why should some, after seeing a man make and set a beautiful porcelain filling, think that they can go and duplicate it, without ever having done any such work before? They are attempting the impossible, and nothing but failure can result. Porcelain is a faithful servant when a due apprecia-tion and mastery of the skill required in its manipulation is understood, but it is just as much of a master when attempted as something easy and not requiring any special effort to become skillful in its use.

Dr. G. H. Wilson, Cleveland: We are to be congratu-lated upon such papers as these presented before our society. I believe their conservatism will voice the best minds of our profession to-day. The use of all new things and ideals will meet with misuse, but this is necessary to estab-lish their proper relation.

I like the distinction the Doctor made between radical enthusiast and conservative enthusiast. Enthusiasts are needed in all walks of life, and it is the radical that brings to the front the more rational conservative enthusiast, and therefore real progress.

We should have thoroughly impressed upon our minds that it is not wise to condemn a process or material because we have met with failures. Perhaps there is no profession in which the personal equation is so prominent a factor in the success or failure as in dentistry; and the use of porcelain being radically different from all former methods, there is little wonder there are many grievous failures; but when a few men have such fine results we should individ-ually apply ourselves to overcome our deficiencies. It is always well to consider if the radical, either for or against, has the manipulative ability or scientific attainments to warrant the position he assumes. The inefficient and conservative should not too severely condemn the radical, because he is needed as a spur to action.

The essayist truly says we are not born successful, and defines for us genius. Some one has said that genius is the ability to do things without hard work. I believe it would be better to say that genius is the power to do things as the result of previous hard work. I do not believe we have a born genius, but we certainly have individuals born with inherent ability to acquire skill and knowledge more readily than others; but it has required months and years of arduous toil for these favored ones to attain unto their degree of perfection.

I would like to have the Doctor explain to what extent rolled gold or platinum plate will absorb moisture under the conditions found in the mouth. It is true that cast metal is more or less porous and that moisture may be forced through metal under high pressure.

I would commend all he has said about the good qualities of porcelain for plate work; but I think he places too much stress upon certain ideas in connection with the retention of artificial denture. I do not believe we should increase the pressure upon any of the vault portion of the mouth, but the pressure should be relieved upon the hard places within the vault. With the alveolar ridge the reverse as true, we increase the pressure upon the soft places. I would make an exception to this rule when there are small abnormally-hard circumscribed portions of the alveolar ridge; the pressure should be relieved upon these. The portion of the alveolar ridge upon which I would especially increase the pressure, is that occupied by the six or eight anterior teeth. In such a case when the denture is placed in the mouth, it would only rest upon the back part of the vault and the anterior portion of the ridge which we are endeavoring to compress. This would result in a large portion of the plate not being in contact with the mucous membrane, producing a partial vacuum, hence the denture is retained by atmospheric pressure. When the soft tissues were compressed the whole of the maxillary portion of the plate would be in contact with the mucous membrane, hence there would be no vacuum space or atmospheric pressure, the denture being retained by adhesion. The one essential is to have uniform pressure, so the denture can not be displaced in mastication.

The essayist speaks of using platinum solder. I agree with Dr. Haskell that this is not necessary. If the two pieces of platinum to be untied are nicely fitted, and a small amount of gold used for solder with high-continued heat, the two metals will be alloyed, and cannot be unsoldered in fusing the porcelain.

I can only partially agree with the Doctor in what he says about open-faced crowns. They should rarely be used, but in a suitable place and properly constructed, they will answer a very excellent purpose. They must be rigidly constructed, or they will cause trouble. A shell crown should never be used upon the anterior teeth excepting in a few cases of old age, when utility may demand them.

I would like to ask the second essayist how it is possible for two men to take the same materials and each produce a porcelain that fuses at the same temperature and yet one will disintegrate in the mouth, and the other will not? It seems to me we are asked to be too credulous. We know that low-fusing porcelain is produced by the addition of potash, and all potash salts are soluble. It is reasonable to suppose that the porcelain will be decomposed by the fluids of the mouth just in proportion to the amount of potash contained. It seems to me it is well for a learned profession to make use of its scientific knowledge and not accept all statements of interested parties.

Dr. J. R. Callahan, Cincinnati: Doctor Goslee has classified men into two general classes—the enthusiast and the skeptical. There is another class, who, for want of a better name, we will call selfish. They are usually skeptical, sometimes cynical and often discouraging. They some times boast of conservatism. When a new idea or appliance appears they save their time, their energy, their money, and let their enthusiastic and wide-awake friend do the work. Then Mr. Selfish slips around and gets Mr. Enthusiast to show him or give him the fruits of his labor, which Mr. Enthusiast is, as a rule, very willing to do notwithstanding the fact that Mr. Selfish seldom says "thank you." In this porcelain work Mr. Selfish is doomed to disappointment —no one can tell him all about it. It is true Mr. Enthusiast can and does help immensely to smooth the road in many places; still, to do porcelain work well, each and every individual will have to work out his own salvation to a greater extent than in any work with which I am familiar, and, as the essayist says, we must each one crawl before we can expect to walk.

Doctor Goslee says truthfully that, among many other requirements, "he should possess the higher ideals of an artist." He means, of course, harmony in color, as well as form and general character. Take the one point of color; we must now study the theory of color with far greater energy and thoroughness than ever before, as for instance some of the circumstances which modify a color. Chevreue says: A given color—red, for instance—may experience many modifications, so as to appear very different from what it really is, according to the circunstances under which it is viewed. It may be modified in its color.

1. By being placed in contact with blue, the red appears yellower.

2. By being in contact with yellow, it appears yellower.

3. By being in contact with green, it appears purer and brighter.

4. By being in contact with black, duller.

5. By being in contact with white, ligher and brighter.

6. By being in contact with grey, brighter.

Thus the same red may appear many different reds according to the circcumstances under which it is viewed.

It may be modified in its intensity or tone; if a dark color be placed beside a different but lighter color, the dark color appears deeper and the light color appears lighter—this the result of contrast of tone. A color is also greatly modified by gloss—as is shown by plumage of birds, the wings of butterflies and by certain flowers. The colors of objects are also greatly modified by the form of the object, which may produce varities of light and shade, and thus exhibit many *tones* of the same color. Both the tone and hue of a colored object are modified by the quality of the light by which it is illuminated—whether it is direct sunlight—diffused daylight—or diffused reflected light.

So we can proceed to the end of the day—and then not have made a start in the study of colors, but it must be done.

The painter with his brush and the paint places upon the canvas the landscape; then the picture is placed upon the wall for inspection—far away from the original scene—viewed by people who never saw the original scene there produced. It may be said by artistic admirers to be very natural, and beautiful, and indeed it may be, but suppose the picture were placed in the immediate vicinity of the original scene and direct comparisons be made, are there many pictures of the very brightest class that will stand that sort of test? Yet that is just what we have to do with our porcelain. It is not enough to say that the porcelain crown or plate or inlay is beautiful, but is it artistic enough to conceal the fact that it is artificial? Is it strong enough to do the work it is intended for? Will the inlay arrest the ravages of decay? Will the presence of this structure disturb the equilibrium of the delicate organs and tissues with which is is surrounded? You will agree with me that these are severe tests, but have we been able to meet these demands?

It has astonished me to see how very quick the public has been to realize the possibilities of porcelain. There is an enormous demand for that which will conceal dental defects. To one who came into the profession on what may be called the gold wave, the change from gold display to porcelain concealment has been very sudden. Along with this sudden change and demand for the artistic is coming an ability to discriminate. As between the artistic and the inartistic, people are going to be able to discriminate much more closely as to results produced by Dr. A and Dr. B. It is quite likely that Dr. A, the inartistic and not overly accurate operator had better let porcelain alone, for porcelain plates, porcelain crowns, porcelain fillings, will show him up most unmercifully. I can speak from the book, for I have poreclain work out that I hope will never fall under your critical eyes. I can now think of some of my productions that are peaches.

We talk continually of high-fusing and low-fusing bodies, of gold and platinum matrix, of direct burnishing of cavity impression, etc. In my opinion no man is thoroughly equipped for porcelain work until he is able to choose the body (high or low-fusing), metal or the method which is best suited to the case in hand; but if I were compelled to select some *one* porcelain body with which to work I should without hestiation select that known as the Brewster bodies and enamels.

Dr. E. S. Fuller, Piqua: First, I want to show you some instruments which I use in the preparation of cavities. They are especially useful in occluso-proximal cavities in molars and bicuspids. I use the smallest size wheel and barrel-shape carborundum points, Nos. 150 and 152. I mount them on worn-out burs. The head of the bur should first be ground square and fitted snugly into the hole in the stone, and then cemented, using Ames' oxy-phosphate of copper cement, which will hold them better than any other cement I have used. Then turn the points up true and to the desired shape in the lathe, using a diamond-point wheel-dresser. They are used to form the outline of the cavity, which is then completed with inverted cone and fissure burs. For occluso-proximal cavities, cut the foil a little longer than its width and a little narrower at one end and clip off each corner. Bend the widest end to an obtuse angle and crease it slightly along its longest axis. Hold in place with pliers and press to place, using hard rolled cotton pellets. These may be used wet if desired, when platinum is used.

Dry cotton alone will complete a matrix for gold, but if platinum, the foil must be pressed to place with burnishers and points.

In making some experiments a few days ago, I observed the fact that if greatest amount of heat is applied at the base of the filled matrix, the tendency toward spheroiding is eliminated, and the body will flow up against the sides of the matrix instead of drawing away and leaving a crevice at the margins. By using this method of melting, the number of fusings will be lessened, and the bubbles usually found in the base of the filling when the foil is removed will not be present. No furnace or blow-pipe at our command will furnish the heat necessary to melt high-fusing porcelain in this way, so we are limited to the use of a low-fusing body. It is to be hoped that some manufacturer will be able to furnish us with an electric furnace that will heat exclusively from below which will enable us to employ this method in our work.

Dr. W. A. Price, Cleveland: I would like to ask Dr. Raymond what his experience has been in observing fillings —inlays— that have been in the mouth a great many years? He has been in a position to see these. This is going to prove a good deal to us, and we would like to have all the information we can get.

Dr. Raymond: Regarding that, I will say that I have had opportunities of observing inlays—porcelain fillings for teeth—a great many years. I was telling Dr. Price this afternoon that I have seen porcelain fillings that had been in the mouth fourteen years, and I have never yet seen decay around them. I have seen the cement with the porcelain broken off, and a great many cases where the cement is dissolved so you could get a thin pointed instrument between the filling and the teeth, and yet there was no decay. I have seen some mouths where other fillings have been replaced three or four times and yet the porcelain fillings were preserving the teeth.

Dr. Hawley: I would like to ask Dr. Raymond what location in the mouth these fillings were? I presume fourteen years ago fillings were placed in different localities from which they are now put in?

Dr. Raymond: Dr. Land was no respecter of location. I might say that the work was not done then as it is done now, or has been done in the last three, four or five years; still Dr. Land is very successful, although he has not always had the patience to do the work in the best manner. He is anxious to get back to the laboratory, and do more experimenting.

Dr. J. B. Beauman, Columbus: It has been said that porcelain was not perfectly impervious to the fluids. I have worked porcelain for about fifty-three years. I have worked all kinds of porcelain, and there are some things about porcelain that I don't know yet. I have tried many experiments, and have had many and funny experiences. I think you all will in the next forty or fifty years. Porcelain any kind of porcelain, is not impervious to the fluids unless it has an absolutely glazed surface, or until the body of the material is brought down to the consistency of glass, which has a glazed surface all over, and you can grind it and it will still be impervious to the fluids; and if you take porcelain of that consistency, and then grind it, it is more or less porous, and so, as has been said, it is impervious.

I have had many porcelain fillings to handle, and I have had great experiences. I have had porcelain fillings that seemed to be well baked, but after a wear of forty years have almost wholly disintegrated and gone to pieces; that is something I could not account for. I have found many porcelain plates that stood intact even longer than that. I had a very funny experience in mending a continuous gum plate. I undertook to mend a continuous gum plate, and it was chipped and cracked, and I went to work and put on some material and put it in the oven, and it appeared to be baking well, and when I took it out the whole body of that continuous gum material that had been put on there turned to powder and came from the teeth as free as though it had never been put there. Washed right off clean. Now, if you can tell why, you can do more than I can.

Dr. W. H. Todd, Columbus: I want to finish that statement by saying that it was one of the plates I made, and I never used anything but Close's body.

Dr. H. L. Ambler, Cleveland: I quite agree with Dr Byram in taking impression of cavities. I believe that in those cases where you have an opportunity to take a correct impression of the cavity it is all right; but where on account of lack of space and the shape of the cavity that you are liable to change the location of your impression in removing it, and as long as that cement remains in there, longer than practical in many cases, it will change relationship and you will have an impression inlay when you attempt to adjust it to the cavity.

Dr. Byram: As I said in my paper, I think the time has not yet come for us to assert ourselves in favor of any one kind of porcelain. Let us try to select the best from all. It makes no difference which form of porcelain one uses if one accomplishes the best results. What may be the best material in one man's hands may not be in the hands of another. Simply because the majority is on one side it does not follow that the majority should specify the materials to be used.

I wish to congratulate Dr. Goslee on his paper. We have discussed both papers since they were read. I think you will find that our ideas are somewhat the same—we try to get the best out of all methods and from all materials. There is one point in Dr. Goslee's paper to which I wish to call attention, the use of porcelain for occlusal surfaces of crowns and bridges. I believe porcelain crowns and bridges give better masticating surfaces than gold crowns or bridges with their occlusal surfaces of gold.

Dr. Callahan: Why?

Dr. Byram: Because gold on the occlusal surface of crowns and bridges wears very smooth and slick, and it is hard to keep food in proper position during the process of mastication. For this reason, inlays are especially applicable in large cavities in molars, and especially in the inferior molars, for esthetic reasons. I construct as many inlays for large cavities in molars as I do for approximal cavities in incisors.

I can hardly agree with Dr. Raymond when he says the time will soon come when all cavities in incisors and cuspids will be filled with porcelain.

There are two points to be considered in all dental operations; first the mechanics, and second, the esthetics. A filling may be ever so well constructed, and the cavity esthetically filled, but if the conditions are such that it will last but a short time, then it is not practical. In many cases on account of the mechanical conditions, I believe it is not best to insert large porcelain fillings in incisors or cuspids.

Dr. Raymond says that high-fusing porcelain is stronger than the Jenkins porcelain enamel. I do not believe the Doctor has thoroughly tested the porcelain, or he would not have made the statement. The Jenkins enamel has the advantage as far as crushing strength is concerned. It is more homogeneous in texture; it is denser and harder. I had an appliance made to test the crushing strength of porcelains. I find that the Brewster enamel will resist a greater force than the foundation or Close's body. Its density is greater than either of the others.

There have been several statements made regarding the transparancy and opacity of porcelain. I seem to have different views from most of you, regarding the transparency of porcelain. When we speak of natural teeth, being transparent, we are drawing the line rather close. While there is some translucency to the teeth, they are not transparent in the sense in which we speak. The opacity of the Jenkins enamel gives it an advantage over the high-fusing porcelains in that the cement will not change the color of the inlay.

Dr. Molyneaux mentions the advantage of taking the glaze off porcelain by grinding, because it gives it a more life-like appearance when placed in the mouth. This is another advantage of the Jenkins enamel over high-fusing. On account of its density it does not take a high glaze like the high-fusing porcelains, and if ground it will take a higher polish.

I can not agree with the Doctor who says the best results are obtained by burnishing the matrix directly into large cavities and then fusing the porcelain without further burnishing. I tried to explain that the larger the mass of porcelain to be fused the greater the tendency of this mass to form a spheroid and the necessity of re-burnishing the matrix in the cavity before the porcelain is extended to the margins.

I have never used enamel rods but have used crushed porcelain. I do not use it to prevent distortion of matrices so much as to control shrinkage. A good way to prevent distortion of the matrix is to make several centers of shrinkage and allow the different portions of porcelain to fuse from different centers.

I can not say from many years of experience just how long inlays will last. I have been constructing them for four years. I am perfectly willing to take the statements of men of reputation and men whose veracity I have no reason to doubt, when they say inlays last for a long time and when they make statements similar to those made in my paper regarding the recurrence of caries. I take it for granted that they know of what they speak.

DR. H. J. GOSLEE, Chicago: In my paper I made an assertion with regard to the hygienic properties of porcelain as compared with gold. I spoke only of the SURFACE, however, if you will remember, and quoted a statement from Dr. Wilbur F. Litch to the effect that in his opinion porcelain was a porous material, and that it did not possess hygienic properties equal to those possessed by a highly-polished surface of gold or platinum. I took exceptions to that statement and made the counter statement that in my opinion the *surface* of a well-vitrified piece of porcelain offered less opportunity for the penetration or absorption of secretions than gold or platinum ever so highly polished. If you grind *beneath* this vitrified surface, however, you do get a structure more porous than metal, but I speak only of a highly-vitrified surface—a surface that has not been ground. The point I made was not so much as relates to the physical properties of either of these substances, but only to mention with some degree of emphasis that the surface of porcelain always remains cleaner in the mouth than does that of any of the metals. I believe that if you will look into it you will find that the surface of porcelain always presents a much more hygienic condition than gold or platinum, and particularly gold.

Dr. Wilson said that he did not give any special preparation to the hard places in the impression and that he paid no attention whatever to the soft places in the mouth. In this connection I feel that the retention and stability of a denture is due altogether to uniform contact with the tissues upon which it rests. To get uniform contact, or an equalization of pressure, we must bear in mind the varying conditions of the tissues upon which the denture is to rest, and to prepare the hard and soft places in the impression and model as to enable us to obtain this.

In speaking about the use of platinum solder in the construction of bridge and crown-work, I think I was taken to task by some one in the discussion as to the advantage of platinum solder over pure gold. The individua parts of all crowns can be soldered with pure gold if they are in absolute contact, and if the gold is properly fused it becomes alloyed with the platinum in such a manner that the parts could not be separated.

I think the object of Dr. Byram's paper has been thoroughly covered. The adoption of *certain* methods is of great importance, and in the minds of those who have not already adopted a certain line of procedure it may be questionable as to which method they should take up and become familiar with. It is my experience, however, that we cannot depend upon any one method. In the adaptation of matrices to simple approximal cavities I prefer to burnish, because you can burnish to an accurate adaptation better and much more quickly than you can take an impression. But in cavities of large proportions, and particularly in posterior teeth, I prefer to take an impression, make the dies, and swage the matrix.

Those who have experimented at all will agree with me in that it is a very difficult matter to closely adapt platinum to large cavities. You can facilitate the adaptation in this class of cases by swaging first, and then after the first bake placing it in the cavity and burnishing directly to its walls.

When considering the relative value of the high and low-fusing bodies, in so far as pertains to strength, I feel that we can get all of the *required strength* with either. I am not prepared to say that the low-fusing bodies possess a greater degree of strength than the high-fusing bodies, because I have not made scientific tests. I agree with the Doctor in that the more homogeneous the mass, the greater the density, but whether density imparts crushing strength or not I am not prepared to say, and hence I would not like to go on record as saying that low-fusing body is in any manner stronger than high-fusing body. I feel sure, however, that either is strong enough for our purpose.

I want to say something in reply to the question as to the permanency of porcelain inlay work. I have seen porcelain inlays made by Dr. Jenkins, which have been in the mouth fourteen years, and while they showed a partial dissolution of some of the ingredients or flux, yet they were doing good service, and caries had not recurred around the margin. In my own work I have inlays that have been doing service four or five years, and are still in a good state of preservation.

Dr. Goslee: I fear from what Dr. Custer said in regard to burnishing that the idea might prevail that I bring the instrument that I burnish with in contact with the matrix, but one should seldom bring the instrument in direct contact with the platinum in burnishing.

Dr. L. E. Custer, Dayton: There was one point brought out in my paper that did not seem to be plain—and that is the use of the pallet of cotton for pushing the matrix in the cavity. It is not the material, it is simply the process of applying the same which will communicate the force fairly equal to all parts—to all margins; it might be anything—it might be the rubber end of a lead pencil. The point was to have some material which would produce an equal force all over and adapt the margins at the same time.

The point has not been brought out, and I will speak of that at this time—that is the etching of the inlay prior to its setting. That is an important point if care is pursued. Make the foundations for inlays of Close material, and then finish with Brewster enamel. The Close material is coarse grained and when etched makes a better retention than if the whole inlay were made of Brewster enamel, or even with Brewster foundation.

In reply to Dr. Fuller who spoke of applying heat to the under surface of the matrix: That is a matter I have not had any actual experience in—don't know just what effect in general practice it has—I can't say; but there is no question but what if heat is applied to the under surface the porcelain begins to fuse there first and gradually as the heat increases the upper surface will be melted, and you would have complete adhesion to the platinum. The question arises whether or not the platinum would not be strained just as much by that as if it were to melt and make an effort to assume a globular form, and leave the matrix at the edges to be filled in at the next baking.

It may be one of the most important points of all of the fundamental things—the adaptation of this which Dr. Fuller has brought out, and it will be one of the first experiments I make.

I believe that burnishing is not the proper way to adapt the matrix to the cavity. It was said in the paper that the minute you apply the burnisher it endows the platinum with life and temper. It is the continued pounding of the body of the metal that puts it in a state of spring, and so it is in the adaptation of the matrix for an inlay. It should not be repeatedly burnished; it should be gently pressed against the walls of the cavity, and if that is done there is no danger that that part of it will go back to its original form. Placing it once against the cavity wall is sufficient, and if you pass a burnisher over it you will find it endowed with life. It should be often annealed.

Now as to the use of rods. I said that the plan which I followed was to select one sufficiently long so when you drop it into the matrix it would lodge against the sides before reaching the bottom.

One of the most practical uses for the porcelain filling is in the large posterior cavities. Some cavities are so large and some distances under the gum as almost to preclude the use of a crown or a filling. It is a simple procedure to take an impression of that, and take it to the laboratory and finish that inlay by the swedging process and returning to the cavity and cementing it in. I think that is the most useful place for the porcelain, in the posterior portion of the mouth.

